# Upper-Limb Motor Recovery in the Late Subacute Phase After Stroke: A Single-Case Experimental Study of Robot-Assisted Therapy, rTMS, and Intensive Training for Upper-Limb Rehabilitation in the First Year After Stroke Using Modern Treatment Strategies

**DOI:** 10.3390/jcm15145564

**Published:** 2026-07-15

**Authors:** Hannu Heikkilä, Aet Ristmägi, Olavi Airaksinen

**Affiliations:** Department of Physical Medicine and Rehabilitation, Kuopio University Hospital, University of Eastern Finland, FI-70210 Kuopio, Finland; olavi.airaksinen@uef.fi (O.A.)

**Keywords:** late subacute stroke, upper-limb motor recovery, robot-assisted therapy, rTMS, intensive training

## Abstract

**Objective**: This study aimed to determine whether robot-assisted therapy, low-frequency rTMS, or intensive therapist-guided upper-limb training provides additional recovery beyond self-directed training in individuals 4–9 months post-stroke. **Design**: A five-phase single-case experimental study was conducted with two baseline phases and three randomized 3-week intervention phases. **Subjects/Patients**: Sixteen adults with moderate-to-severe upper-limb motor impairment in the late subacute phase after stroke (4–9 months post-stroke) were included. **Methods**: Participants completed intensive task-oriented training, low-frequency rTMS, and robot-assisted therapy in a randomized order. Primary outcomes were Fugl–Meyer Assessment for the Upper Extremity (FMA-UE), active range of motion, and muscle strength. Secondary outcomes included EQ-5D and WHODAS 2.0. **Results**: FMA-UE motor scores improved significantly over the study period (8–10 points). However, comparable gains occurred during baseline phases. rTMS and intensive training produced within-phase improvements, whereas robotic therapy did not. Participants with higher initial FMA-UE scores improved, while those with severe paresis showed minimal benefit and occasional decline during rTMS. Disability and quality-of-life measures remained stable. Gains were maintained at 1-year follow-up. **Conclusions**: In the late subacute phase after stroke, modest upper-limb motor improvements occurred, but effects were not clearly attributable to specific interventions beyond ongoing recovery. Treatment response depended strongly on baseline motor severity, with limited benefit in severe paresis.

## 1. Introduction

Stroke remains one of the leading global causes of long-term disability, imposing substantial healthcare and socioeconomic burdens. Approximately 50% of stroke survivors experience persistent upper-limb motor impairment [1], which significantly limits reaching, grasping, object manipulation, and independence in activities of daily living [2]. Consequently, upper-limb paresis is a key determinant of functional outcome and quality of life after stroke.

Post-stroke recovery follows a nonlinear trajectory. The majority of spontaneous neurological recovery occurs within the first three months, with a plateau typically reached by six months [3,4]. This early spontaneous recovery may confound the interpretation of treatment effects in the acute phase [5]. However, accumulating evidence indicates that neuroplastic processes continue beyond this period, extending into the late subacute and chronic phases, thereby supporting the rationale for continued rehabilitation during the first year after stroke [6,7,8].

Recent advances in neurorehabilitation have focused on interventions that enhance neuroplasticity and increase the intensity and specificity of training. Robot-assisted upper-limb therapy enables high-repetition, task-specific practice and has demonstrated modest improvements in arm function, muscle strength, and activities of daily living [9,10]. In parallel, non-invasive brain stimulation techniques such as repetitive transcranial magnetic stimulation (rTMS) have been investigated as adjunctive therapies. Low-frequency rTMS applied to the contralesional primary motor cortex (M1) is hypothesized to reduce maladaptive interhemispheric inhibition and facilitate motor recovery [11,12]. Nevertheless, evidence regarding its effectiveness in the late subacute phase remains inconsistent, and relatively few studies have employed standardized outcomes such as the Fugl–Meyer Assessment for the Upper Extremity (FMA-UE) [13]. In addition, in Scandinavian clinical practice, rTMS is not routinely implemented in neurorehabilitation due to limited availability and ongoing uncertainty regarding optimal stimulation protocols and patient selection.

To address these gaps, the present study employed a sequential crossover design with repeated within-subject measurements to evaluate and compare multiple upper-limb rehabilitation interventions. This design enables each participant to serve as their own control, facilitating the assessment of intervention-specific effects while minimizing inter-individual variability.

The primary aim of this study was to compare the effects of different rehabilitation interventions relative to baseline within individuals. The secondary aim was to examine whether robot-assisted therapy, low-frequency rTMS, and therapist-guided intensive training result in clinically meaningful improvements in upper-limb function in individuals 4–9 months post-stroke, a period often characterized by a clinical plateau following initial subacute rehabilitation.

## 2. Methods

### 2.1. Study Design

This study employed a sequential crossover design with repeated within-subject measurements to evaluate and compare the effects of multiple upper-limb rehabilitation interventions. In this design, all participants were exposed to each intervention condition in a randomized sequence, allowing each individual to serve as their own control. This approach minimizes inter-individual variability and enables direct within-subject comparison of treatment effects, which is particularly advantageous in neurorehabilitation research [14,15].

**Figure 1:**
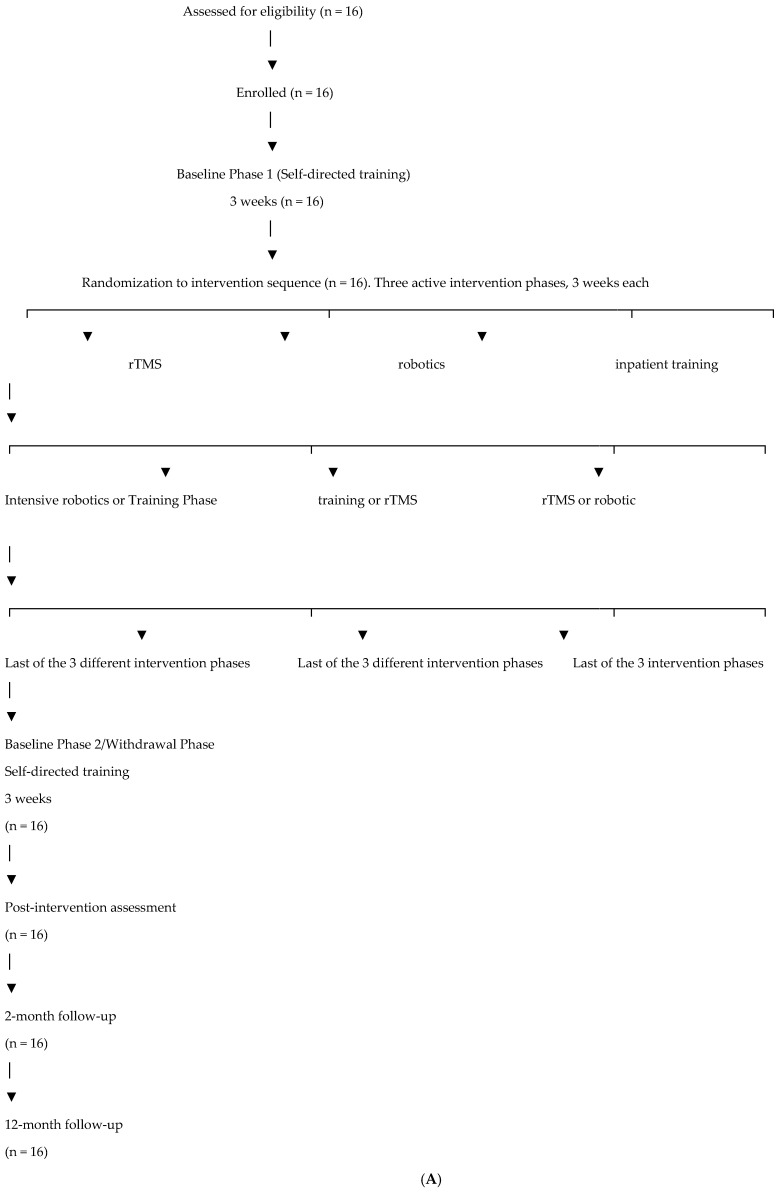

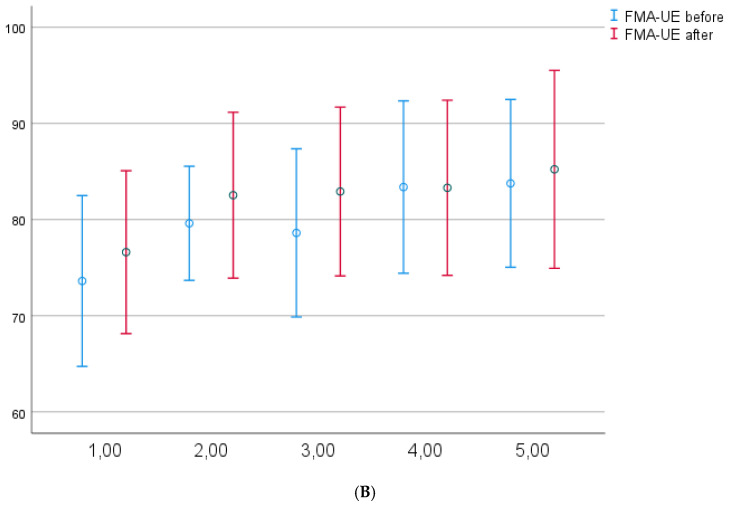
(**A**) Flow of participants through a sequential crossover design with repeated within-subject measurements to examine the effects of three contemporary rehabilitation modalities—robot-assisted therapy, low-frequency repetitive transcranial magnetic stimulation (rTMS), and intensive therapist-guided motor training—on upper-limb recovery. All participants had completed intensive inpatient rehabilitation and were enrolled at the point of clinical plateau, defined as the absence of measurable improvement on serial weekly Functional Independence Measure (FIM) assessments. (**B**) Fugl–Meyer UE total scores before and after treatment for different baseline phases. Baseline 1 = with no active treatment; 2 = rTMS phase; 3 = intensive inpatient training; 4 = robotic training; 5 = baseline 2 without active treatment.

1. The study protocol consisted of five sequential phases, each lasting three weeks, resulting in a total intervention period of 15 weeks (Figure 1A). Baseline 1 (A)
  Participants underwent a 3-week baseline period consisting of self-directed upper-limb training in addition to usual care. This phase was used to establish an individual reference level of performance and to assess baseline stability.
2. Intervention Phases (B, C, D)
  Three active intervention conditions were administered, each for 3 weeks, in a randomized order:
  ∘ **B**: low-frequency repetitive transcranial magnetic stimulation (rTMS);
  ∘ **C**: intensive therapist-guided inpatient upper-limb motor training;
  ∘ **D**: robot-assisted upper-limb therapy.
  This crossover structure enabled within-subject comparisons across intervention conditions, reducing the influence of confounding factors related to between-subject variability.
3. Withdrawal Phase/Baseline Phase 2 (E)
  A final 3-week phase involved self-directed training only, without structured therapeutic interventions. This phase was included to explore the maintenance or attenuation of treatment effects following the withdrawal of active interventions.
4. Follow-up Assessments
  Outcome measures were reassessed at 2 months post-intervention and 12 months to evaluate the durability of treatment effects over time.
5. Baseline 1 (A)
  Participants underwent a 3-week baseline period consisting of self-directed upper-limb training in addition to usual care. This phase was used to establish an individual reference level of performance and to assess baseline stability.
6. Intervention Phases (B, C, D)
  Three active intervention conditions were administered, each for 3 weeks, in a randomized order:
  ∘ **B**: low-frequency repetitive transcranial magnetic stimulation (rTMS);
  ∘ **C**: intensive therapist-guided inpatient upper-limb motor training;
  ∘ **D**: robot-assisted upper-limb therapy.
  This crossover structure enabled within-subject comparisons across intervention conditions, reducing the influence of confounding factors related to between-subject variability.
7. Withdrawal Phase/Baseline Phase 2 (E)
  A final 3-week phase involved self-directed training only, without structured therapeutic interventions. This phase was included to explore the maintenance or attenuation of treatment effects following the withdrawal of active interventions.
8. Follow-up Assessments
  Outcome measures were reassessed at 2 months post-intervention and 12 months to evaluate the durability of treatment effects over time.

### 2.2. Outcome Assessment

Outcome measures were collected at the beginning and at the end of each phase, resulting in repeated measurements across all study conditions. This allowed for detailed evaluation of within-phase changes and between-phase differences in upper-limb function. 

Additional Clarifications:

- During the study period, participants were followed within a structured outpatient care program, and no additional rehabilitation interventions were permitted, ensuring that observed effects were attributable to the study conditions.
- The baseline and withdrawal phases served as reference conditions, although no formal washout period was implemented due to the expected persistence of rehabilitation effects. This is acknowledged as a limitation.

### 2.3. Ethical Considerations

The study was approved by the regional ethics committee (SATSHP/697/13.01/2020). All participants provided written informed consent prior to enrollment. The trial was registered at ClinicalTrials.gov (NCT05520489).

### 2.4. Participants

Sixteen participants (mean age 61.3 years, SD = 12.8) were recruited from the inpatient rehabilitation ward and outpatient clinic at Satasairaala (Table 1). All participants had completed intensive inpatient rehabilitation and were enrolled at the point of clinical plateau, defined as the absence of measurable improvement on serial weekly Functional Independence Measure (FIM) assessments.

The sample size of 16 participants was determined based on the following:

- Feasibility constraints, including time, resources, and availability of advanced rehabilitation technologies;
- The intensive and time-demanding nature of the intervention protocol;
- Recommendations for pilot studies, aimed at estimating variability and informing the design of future, adequately powered trials.

### 2.5. Inclusion Criteria

- Age 18–80 years;
- Ischemic or hemorrhagic stroke occurring 2–9 months prior to enrollment;
- Presence of hemiparesis or hemiplegia;
- Moderate-to-severe upper-limb impairment as assessed by the Fugl–Meyer Assessment for Upper Extremity (FMA-UE);
- Stabilized spontaneous recovery at the time of inclusion.

### 2.6. Exclusion Criteria

- Contraindications to transcranial magnetic stimulation (TMS) [16];
- Significant cognitive or language impairments preventing participation;
- Unstable neurological or medical conditions.

### 2.7. Interventions

#### 2.7.1. Repetitive Transcranial Magnetic Stimulation (rTMS)

Participants received 15 weekday sessions of low-frequency (1 Hz) rTMS applied to the contralesional primary motor cortex (M1), using a Magstim Rapid stimulator with Visor2 neuronavigation (The Magstim Company Ltd, Whitland, United Kingdom). Stimulation intensity was set at 80–90% of the resting motor threshold (rMT). Each session delivered 1200 pulses (600 + 600 pulses separated by a 10 min interval). The rMT was reassessed every 2–3 sessions to ensure appropriate stimulation intensity.

#### 2.7.2. Robot-Assisted Upper-Limb Therapy

Robot-assisted therapy was delivered using the Tyromotion Diego and Pablo systems. Participants completed four 60 min sessions per week for 3 weeks. Training included:

- Weight-supported three-dimensional reaching tasks;
- Fine motor exercises;
- High-repetition practice (approximately 150–400 repetitions per session);
- Standardized, task-oriented virtual training modules (e.g., *Swimming*, *Shooting*, *Ship*, *Apple Orchard*).

#### 2.7.3. Intensive Therapist-Guided Motor Training

Therapist-guided inpatient training consisted of task-oriented, high-intensity exercises tailored to individual functional goals. Each 3-week intervention phase included:

- Three sessions per week focusing on fine motor and complex functional tasks;
- One session per week emphasizing proximal and multi-joint movements;
- Each session lasted 60 min.

### 2.8. Primary Outcomes

- Fugl–Meyer Assessment for Upper Extremity (FMA-UE) [17,18,19,20], total score range 0–126, including motor function (0–66), sensation (0–12), passive joint motion (0–24), and joint pain (0–24).
- Active joint range of motion (ROM) assessed using the Tyromotion Diego and Pablo systems.
- Muscle strength, assessed using the same digital systems.

### 2.9. Secondary Outcomes

- EQ-5D for health-related quality of life;
- WHODAS 2.0 for disability assessment.

### 2.10. Statistical Analysis

Statistical analyses were performed using SPSS version 28.0 (IBM Corp., 2021). Statistical significance was set at *p* < 0.05. Given the within-subject repeated-measures crossover design and small sample size, nonparametric methods were primarily applied. A Friedman test was used to evaluate overall differences across study phases within individuals. Wilcoxon signed-rank tests were conducted as post hoc analyses for pairwise within-subject comparisons between phases. For variables meeting assumptions of normality, repeated-measures analysis of variance (ANOVA) was used to assess differences across study phases. In addition, participants were stratified into subgroups based on baseline Fugl–Meyer Assessment for Upper Extremity (FMA-UE) scores (e.g., lower vs. higher motor function). To explore whether baseline impairment level influenced treatment response, between-group comparisons were performed using the Mann–Whitney U test for nonparametric data. For normally distributed variables, independent-samples *t*-tests were applied.

These subgroup analyses were exploratory and aimed to assess potential differences in responsiveness to interventions according to baseline motor impairment.

## 3. Results

### 3.1. Fugl–Meyer Assessment (FMA-UE)

**Figure 2:**
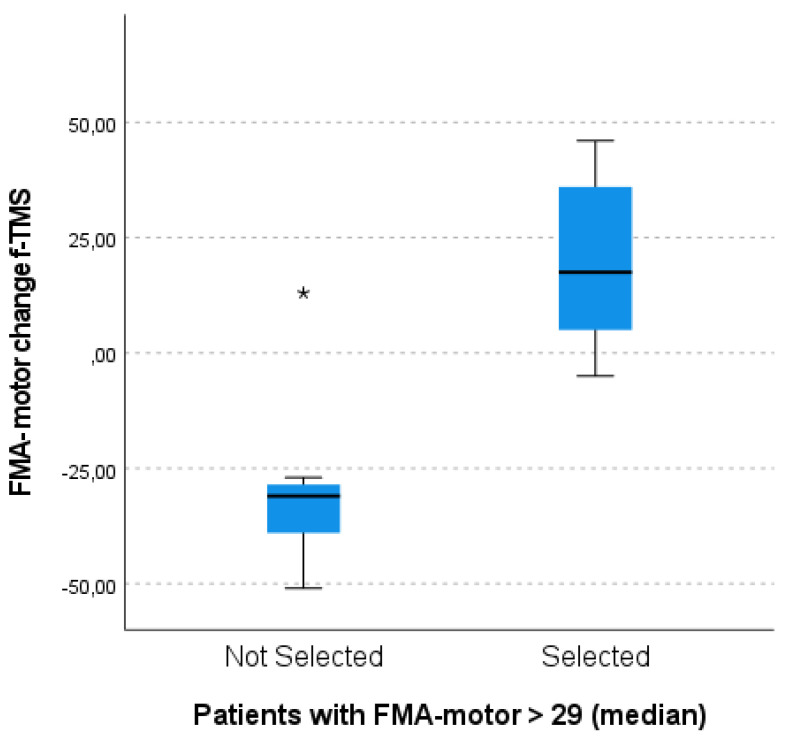
Fugl–Meyer motor scale change under r TMS-therapy phase for patients with FMA motor < 29 (median) and FM motor > 29. Significant differences occurred for the r-TMS phase (*p* < 0.001). * in the figure shows an outlier.

Fugl–Meyer Assessment for Upper Extremity (FMA-UE) scores showed a progressive increase over the rehabilitation timeline (Figure 1), with the largest changes observed in the motor subscale (Table 2). A Friedman test revealed no statistically significant differences between intervention phases for motor subscale (χ^2^(2) = 1.60, *p* = 0.81), passive joint movement (χ^2^(2) = 4.02, *p* = 0.40), joint pain (χ^2^(2) = 5.31, *p* = 0.26), and total FMA-UE score (χ^2^(2) = 4.72, *p* = 0.32) (Figure 2). However, a significant difference across repeated measurements was observed for the sensory subscale (χ^2^(2) = 12.53, *p* = 0.014). Post hoc pairwise comparisons using Wilcoxon signed-rank tests with Bonferroni correction indicated a trend toward greater improvement during the rTMS phase compared with intensive training (*p* = 0.06) and robot-assisted therapy (*p* = 0.12), although these differences did not reach statistical significance after correction.

Across all phases, post-intervention scores consistently exceeded pre-intervention values (Figure 1). Improvements were observed not only during active intervention phases but also during baseline periods, suggesting a continued recovery trajectory independent of specific interventions.

Motor function demonstrated the most pronounced improvements, with statistically significant within-phase individual gains observed during intensive training, rTMS, and the first baseline phase (Table 3). In contrast, sensory function and passive joint movement showed only modest changes, and joint pain remained largely stable throughout the study. Notably, only the rTMS phase was associated with a statistically significant within-phase improvement in the sensory subscale (Table 3), although the overall magnitude of sensory recovery remained limited.

### 3.2. Differences by Baseline FMA–UE Score

Mean upper-extremity Fugl–Meyer scores increased steadily along the rehabilitation trajectory prior to study enrollment, rising from 60 at admission to the specialized rehabilitation ward to 69 at discharge from primary rehabilitation, to 74 at the start of the study, 84 at the end of the study, and 85 at 1-year follow-up.

### 3.3. Differences by Baseline FMA-Motor Function

Mean FMA-UE scores increased steadily prior to study enrollment, from 60 at admission to 69 at discharge from primary rehabilitation and 74 at study baseline, reaching 84 at study completion and 85 at 1-year follow-up, indicating an overall recovery trajectory. When stratified by baseline motor impairment (median FMA motor score = 29), between-group analysis revealed differential responses to interventions. Participants with lower baseline motor function demonstrated a significant decline in motor performance during the rTMS phase (F = 23.7, p < 0.001) (Figure 2), whereas those with higher baseline function did not show this pattern. Clinically meaningful improvements in FMA motor and sensory subscores were observed primarily among participants with baseline FMA-UE scores above 69 (median) (Table 4). In contrast, individuals with more severe impairment exhibited minimal or no measurable improvement across intervention phases. At 1-year follow-up, no significant differences were observed compared with post-intervention scores (Table 5), suggesting limited long-term additional gains following completion of the intervention phases.

### 3.4. Joint Movement (ROM)

**Figure 3:**
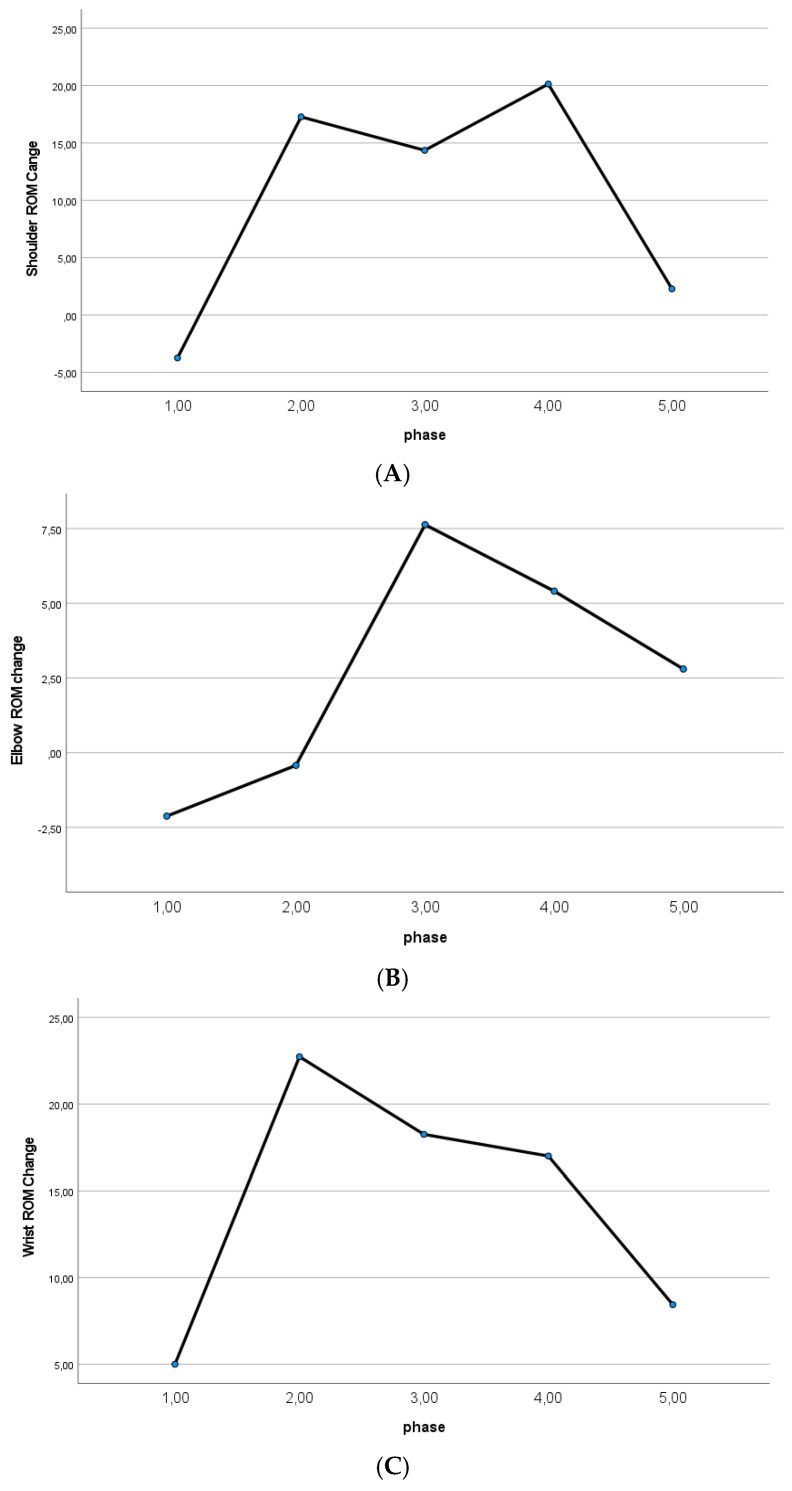
Changes in total range of movement under different phases for (**A**) shoulder joint, (**B**) elbow joint, and (**C**) wrist joint. Phases were 1 = baseline, 2 = TMS, 3 = active inpatient training, 4 = robotics training, and 5 = baseline 2.

Only small improvements in active joint range of motion (aROM) were observed across the study period (Figure 3A–C).

A Friedman test showed no statistically significant differences between intervention phases for shoulder ROM (χ^2^(2) = 5.42, *p* = 0.25), elbow ROM (χ^2^(2) = 3.47, *p* = 0.48), or wrist ROM (χ^2^(2) = 8.80, *p* = 0.07). The largest improvements occurred during active intervention phases, whereas both baseline phases demonstrated little to no measurable change (Table 6). Overall, the magnitude of change in joint mobility remained modest.

### 3.5. Hand Function

**Figure 4:**
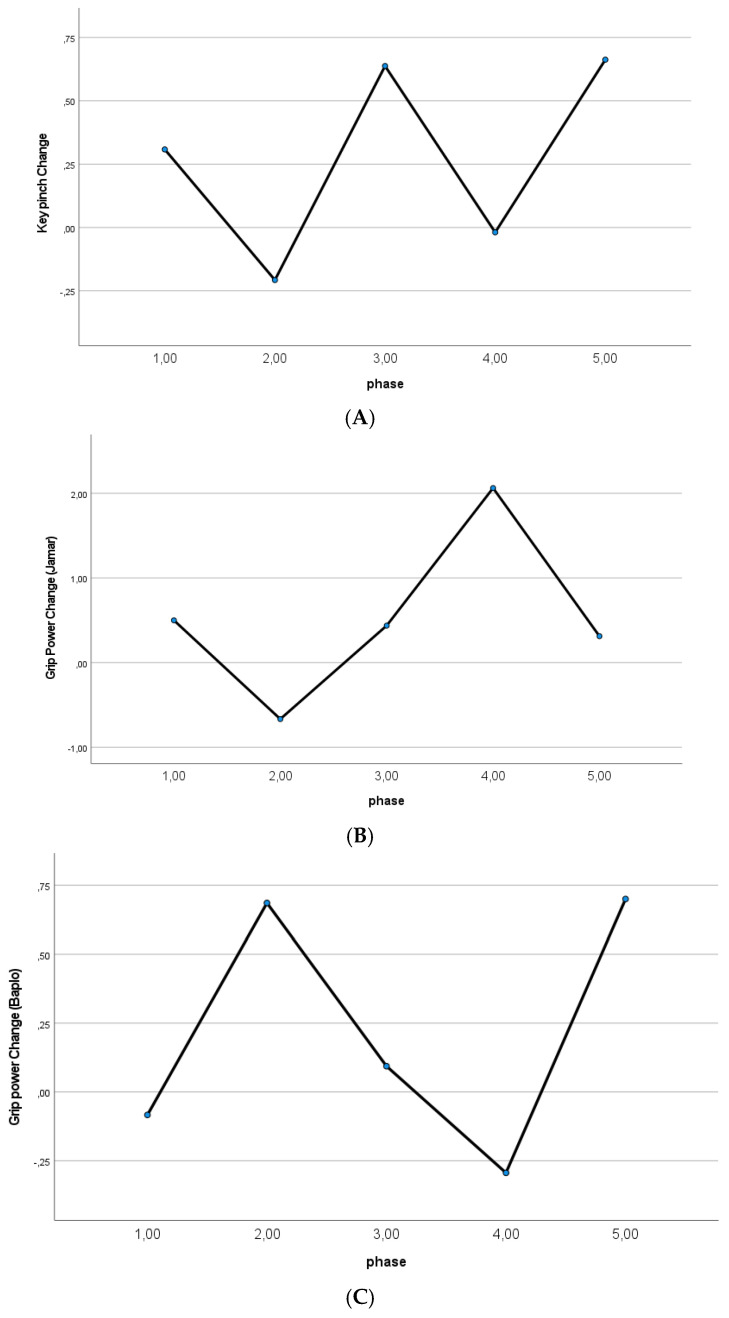
Changes under different phases for (**A**) key pinch strength, (**B**) hand grip strength measured by Jamar, and (**C**) hand grip strength measured by Pablo. Phases were 1 = baseline, 2 = TMS, 3 = active inpatient training, 4 = robotics training, and 5 = baseline 2.

No statistically significant differences between intervention phases were observed for hand function measures: key pinch strength (χ^2^(2) = 5.88, *p* = 0.21), grip strength (Jamar dynamometer) (χ^2^(2) = 3.19, *p* = 0.53), and grip strength (Pablo system) (χ^2^(2) = 1.70, *p* = 0.79). Overall, only modest improvements in hand function were observed across study phases, with no intervention demonstrating consistently superior effects (Figure 4A–C).

## 4. Discussion

This study employed a sequential crossover design with repeated within-subject measurements to examine the effects of three contemporary rehabilitation modalities—robot-assisted therapy, low-frequency repetitive transcranial magnetic stimulation (rTMS), and intensive therapist-guided motor training—on upper-limb recovery in the late subacute to early chronic phase after stroke. Overall, modest improvements in motor performance were observed over time, but these changes did not consistently correspond to specific intervention phases. Notably, similar improvements were also observed during baseline periods without active treatment, suggesting that residual spontaneous recovery, repeated testing effects, or other non-specific factors likely contributed to the observed gains.

A key finding was that baseline motor impairment strongly influenced treatment response. Participants with higher baseline Fugl–Meyer Assessment for Upper Extremity (FMA-UE) scores (≥69 median) demonstrated small but measurable improvements across intervention phases, whereas individuals with more severe impairment showed minimal benefit. Importantly, participants with the lowest baseline FMA-motor function (<29 median) exhibited a significant decline in motor performance during the rTMS phase. This finding contrasts with previous studies suggesting that low-frequency rTMS over the contralesional primary motor cortex may facilitate recovery by reducing maladaptive interhemispheric inhibition [11,12]. One possible explanation is that, in individuals with severe paresis, further suppression of contralesional cortical activity may reduce compensatory recruitment mechanisms, thereby negatively affecting motor performance. In contrast, participants with better preserved motor function appeared more likely to benefit from rTMS, consistent with evidence that intact corticospinal pathways and residual voluntary control are key determinants of responsiveness to neuromodulation.

Although overall FMA-UE scores improved over time, the lack of significant differences between intervention phases limits the ability to attribute these gains to any specific therapy. The mean improvement of approximately 8–10 points in FMA-UE scores is within the range considered clinically meaningful and is comparable to previously reported recovery trajectories in the subacute phase. However, such improvements are also consistent with expected recovery under usual care, highlighting the difficulty of disentangling intervention effects from ongoing biological recovery processes at this stage.

Phase-specific analyses indicated that rTMS and intensive therapist-guided training were associated with within-phase improvements, whereas robot-assisted therapy showed more limited effects. However, the presence of significant improvement during the initial baseline phase suggests that time-dependent recovery processes were still active at study entry. The absence of further significant change during the second baseline phase may indicate a gradual plateau in recovery, although this process is likely heterogeneous across individuals.

Baseline severity emerged as a consistent moderator of outcomes. Participants with higher initial motor function demonstrated improvements in both motor performance and active range of motion, whereas those with more severe impairment showed little or no change. This finding aligns with existing literature emphasizing the importance of baseline function, corticospinal tract integrity, and overall stroke severity as predictors of recovery potential [21,22,23,24,25,26,27,28,29,30,31,32,33]. The limited responsiveness observed in individuals with severe paresis underscores the need for alternative or adjunctive rehabilitation strategies in this subgroup, including assistive technologies, compensatory approaches, or interventions specifically targeting profound motor impairment.

Improvements were primarily observed in motor control and active movement, particularly at the shoulder and wrist. In contrast, passive joint mobility and pain remained largely unchanged, suggesting that the observed gains were driven by improved motor execution rather than structural changes or reductions in pain. Pain levels were low at baseline, limiting the potential for detectable change.

Measures of global disability, participation, and health-related quality of life remained stable throughout the study period. This highlights the well-recognized disconnect between improvements in isolated motor capacity and broader functional outcomes, which are influenced by multiple physical, cognitive, and environmental factors.

Follow-up assessments at 1 year indicated that the observed functional gains were largely maintained over time, with no significant decline in FMA-UE scores, range of motion, or strength measures. This suggests that improvements achieved during the intervention period reflect stable adaptations rather than transient effects.

Several limitations should be considered. First, the small sample size and pilot nature of the study limit statistical power and generalizability. Second, heterogeneity in lesion characteristics, baseline impairment, and time since stroke may have influenced treatment responsiveness. Third, the absence of a parallel control group limits causal inference, and baseline phases cannot fully account for spontaneous recovery. Fourth, the lack of washout periods introduces the possibility of carryover effects between intervention phases. Additionally, the relatively short duration of each intervention phase may have been insufficient to elicit robust between-phase differences. Although individual single-case analyses using piecewise regression may provide additional information regarding patient-specific treatment trajectories, such analyses were beyond the scope of the present study and were not expected to alter the main conclusion that no consistent differences were observed among the three active rehabilitation strategies. Future studies with larger samples should consider mixed-effects or regression-based models to account for baseline differences and relevant covariates.

Finally, the absence of neurophysiological biomarkers (e.g., motor evoked potentials, measures of interhemispheric inhibition, or imaging-based markers of corticospinal tract integrity) limits mechanistic interpretation and the ability to identify responders to specific interventions.

## 5. Conclusions

In this pilot study, upper-limb rehabilitation delivered during the late subacute phase after stroke was associated with modest but clinically meaningful improvements in motor function. However, robot-assisted therapy, low-frequency rTMS, and intensive therapist-guided training did not demonstrate clear superiority over baseline conditions, suggesting that observed gains may largely reflect ongoing recovery processes. Treatment response appeared to depend on baseline motor function, with individuals with higher initial function showing greater benefit, while those with severe impairment demonstrated limited or even negative responses, particularly during contralesional inhibitory rTMS [34].

These findings underscore the importance of patient stratification and individualized rehabilitation approaches. Larger controlled studies incorporating neurophysiological markers and stratification by baseline severity are needed to better identify responders and optimize treatment selection. Despite improvements in upper-limb outcomes, broader measures of disability, participation, and quality of life remained unchanged, highlighting the multifactorial nature of post-stroke recovery.

## 6. Clinical Implications

This study suggests that patients with moderate residual motor function appear more likely to benefit from active, technology-enhanced interventions, whereas individuals with severe paresis may require alternative strategies focused on compensation, assistive technologies, and caregiver support. Clinicians should therefore tailor rehabilitation intensity and modality based on baseline motor severity rather than applying uniform protocols.

## 7. Key Findings

These results suggest limited potential for post-discharge upper-limb rehabilitation to produce functional gains in individuals with moderate impairment once inpatient recovery has plateaued.

## 8. Clinical Trial Registration Statement

This study was approved by the Regional Ethics Committee of Satakunta Hospital District, Finland (approval number SATSHP/697/13.01/2020). All participants provided written informed consent prior to participation in accordance with the Declaration of Helsinki.

The trial was prospectively registered at ClinicalTrials.gov (Identifier: NCT05520489). The study employed a randomized sequential crossover design in which all participants received each intervention condition in a randomized order. Participants were enrolled between the late subacute stages of stroke recovery (4–9 months post-stroke) and completed two baseline/self-directed training phases and three active intervention phases consisting of low-frequency repetitive transcranial magnetic stimulation (rTMS), robot-assisted therapy, and intensive therapist-guided upper-limb training.

The authors confirm that all ongoing and related trials for this intervention are registered and that the study was conducted according to the approved protocol.

## Figures and Tables

**Table 1:** Baseline characteristics of participants.

Variable	n	Mean (SD)/%
Age (years)	16	61.3 (13.9)
Sex		
**Men**	7	44%
**Women**	9	56%
Paresis Side		
**Right**	10	62%
**Left**	6	38%
FMA-UE		
**Total motor function**	15	27.3/66
**sensation**	15	7.0/12
**passive joint motion**	15	20.8/24
**joint pain**	15	20.7/24
**mRS**		
**Admission**	16	4.2 (Median) 4
**Discharge**	16	3.2 (Median) 3
**1 month**	16	2.9 (Median) 3
**6 months**	16	2.7 (Median) 2
**1 year**	16	2.6 (Median) 2
EQ-5D HRQoL		
**Discharge**	15	0.43
**1 month**	10	0.52
**6 months**	13	0.50
**1 year**	13	0.52

**Table 2:** Total individual changes in outcome measures over the entire study period.

Outcome Measure	Time Point	Mean	n	SD	Z	*p* Value
FMA–Motor	Baseline	27.3	15	18.4	2.9	**<0.001**
	Post-intervention	35.8	15	20.6		
FMA—Sensory	Baseline	7.0	13	4.3	2.1	**0.03**
	Post-intervention	8.7	13	4.1		
FMA—Joint Sensation	Baseline	20.8	13	3.2	−1.3	0.19
	Post-intervention	19.6	13	3.0		
FMA—Joint Pain	Baseline	20.8	13	2.4	0.05	0.96
	Post-intervention	20.8	13	3.7		
Fugl–Meyer Total Score	Baseline	73.6	14	21.8	2.2	**0.03**
	Post-intervention	83.7	14	23.6		
Shoulder ROM—Flexion (°)	Baseline	73.7	14	66.8	1.7	0.09
	Post-intervention	88.6	14	66.6		
Shoulder ROM—Abduction (°)	Baseline	51.5	14	57.3	2.8	<0.006
	Post-intervention	81.7	14	66.6		
Shoulder ROM—Rotation (°)	Baseline	33.8	15	30.1	1.5	0.14
	Post-intervention	40.5	15	30.0		
Elbow ROM—Flexion (°)	Baseline	93.2	15	42.0	1.5	0.13
	Post-intervention	107.7	15	40.3		
Elbow ROM—Extension (°)	Baseline	8.7	14	16.4	0.73	0.46
	Post-intervention	8.5	14	18.1		
Wrist ROM—Palmar Flexion (°)	Baseline	21.7	15	27.1	2.1	**0.04**
	Post-intervention	31.8	15	27.7		
Wrist ROM—Dorsal Extension (°)	Baseline	29.7	15	27.6	1.7	0.08
	Post-intervention	37.9	15	27.7		
Wrist ROM—Ulnar Deviation (°)	Baseline	16.0	16	18.2	1.8	0.07
	Post-intervention	20.6	16	18.9		
Wrist ROM—Radial Deviation (°)	Baseline	6.0	16	9.1	2.2	**0.03**
	Post-intervention	12.2	16	9.7		
Grip Strength (kg) Jamar	Baseline	3.6	14	4.5	2.3	**0.02**
	Post-intervention	4.5	14	4.5		
Key Pinch Strength (kg)	Baseline	1.4	14	2.0	2.5	**0.01**
	Post-intervention	2.7	16	2.2		
Compressive Force (kg) Pablo	Baseline	4.6	15	6.2	1.7	0.09
	Post-intervention	6.5	15	6.5		
Pain (VAS)	Baseline	1.1	14	1.9	1.6	0.10
	Post-intervention	2.5	14	4.0		

Abbreviations: FMA = Fugl–Meyer Assessment; ROM = range of motion; VAS = Visual Analogue Scale.

**Table 3:** Within-phase changes in Fugl–Meyer Assessment for Upper Extremity (FM-UE) subscores across treatment phases.

Outcome	Phase	Time Point	Mean	n	SD	*p* Value
FM-UE Motor	Baseline 2	Before	35.9	16	19.9	0.14
		After	37.0	16	20.5	
	Robotic Therapy	Before	34.6	16	20.3	0.14
		After	35.6	16	20.7	
	rTMS	Before	32.7	16	17.7	**0.05**
		After	34.6	16	19.9	
	Intensive Training	Before	32.6	16	19.3	**0.006**
		After	34.8	16	19.2	
	Baseline 1	Before	27.3	15	18.4	**0.04**
		After	30.7	15	18.3	
FM-UE Sensory	Baseline 2	Before	8.4	14	4.5	0.59
		After	8.7	14	4.0	
	Robotic Therapy	Before	8.3	14	4.0	0.55
		After	8.4	14	4.3	
	rTMS	Before	7.4	14	4.4	**0.05**
		After	8.5	14	4.1	
	Intensive Training	Before	8.4	14	4.4	0.41
		After	8.1	14	4.4	
	Baseline 1	Before	7.0	13	4.9	*0.06*
		After	7.8	13	4.29	
FM-UE Passive	Baseline 2	Before	19.5	13	2.99	0.64
Joint Movement		After	19.9	13	3.1	
	Robotic Therapy	Before	20.5	13	2.7	0.18
		After	19.3	13	3.1	
	rTMS	Before	19.8	13	2.8	0.27
		After	20.3	13	2.8	
	Intensive Training	Before	19.3	13	2.6	**0.03**
		After	20.1	13	2.1	
	Baseline 1	Before	20.8	13	3.3	0.38
		After	20.1	13	3.0	

Abbreviations: FM-UE = Fugl–Meyer Assessment for Upper Extremity; rTMS = repetitive transcranial magnetic stimulation.

**Table 4:** Fugl–Meyer score outcomes for patients with scores higher than median and lower than median in the study (Low FM (<69), High FM (≥69).

Outcome	Group	Mean	SD	*p* Value
Fugl–Meyer Total Score (Pre-intervention)	Low FM (<69)	65.0	17.4	<0.001
	High FM (≥69)	95.0	14.9	
Fugl–Meyer Total Score (Post-intervention)	Low FM	60.9	14.0	<0.001
	High FM	101.8	9.0	
FM Motor Change	Low FM	−4.5	15.5	0.002
	High FM	6.7	12.6	
FM Sensory Change	Low FM	7.1	9.6	<0.001
	High FM	40.7	6.5	
FM ROM Change	Low FM	−0.3	1.7	0.43
	High FM	0.1	2.1	
FM Pain Change	Low FM	0.0	2.5	0.85
	High FM	0.1	1.7	
FM Total Change	Low FM	−4.2	15.1	<0.001
	High FM	7.2	12.6	
AROM Shoulder Flexion Change (°)	Low FM	2.6	17.4	0.21
	High FM	8.5	22.0	
AROM Shoulder Abduction Change (°)	Low FM	2.8	14.9	0.24
	High FM	9.0	26.4	
AROM Shoulder Rotation Change (°)	Low FM	−0.5	11.2	0.44
	High FM	1.6	10.4	
Total Shoulder AROM Change (°)	Low FM	4.9	33.3	0.16
	High FM	19.1	46.4	
AROM Elbow Flexion Change (°)	Low FM	4.7	28.2	0.35
	High FM	0.0	3.6	
AROM Elbow Extension Change (°)	Low FM	1.1	7.2	0.38
	High FM	0.0	0.0	
Total Wrist AROM Change (°)	Low FM	2.4	9.9	<0.001
	High FM	22.7	23.4	
Total Upper-Extremity AROM Change (°)	Low FM	13.8	48.8	0.04
	High FM	43.2	58.1	
mRS (Pre-intervention)	Low FM	3.3	0.49	0.09
	High FM	2.6	0.82	
mRS (6-month Follow-up)	Low FM	3.0	0.82	0.32
	High FM	2.3	1.0	
mRS (12-month Follow-up)	Low FM	3.0	1.0	0.54
	High FM	2.6	0.9	
WHODAS (Pre-intervention)	Low FM	0.68	0.10	0.18
	High FM	0.82	0.17	
WHODAS (6-month Follow-up)	Low FM	0.75	0.13	0.32
	High FM	0.83	0.12	
WHODAS (12-month Follow-up)	Low FM	0.74	0.11	0.54
	High FM	0.80	0.18	
EQ-5D Index (Pre-intervention)	Low FM	0.56	0.21	0.63
	High FM	0.49	0.15	
EQ-5D Index (6-month Follow-up)	Low FM	0.37	0.11	0.12
	High FM	0.51	0.15	
EQ-5D Index (12-month Follow-up)	Low FM	0.43	0.15	0.69
	High FM	0.48	0.26	

**Table 5:** Individual changes in outcome measures from the end of the treatment period to 1-year follow-up.

Outcome Measure	Time Point	N	Mean	SD	*p* Value
Fugl–Meyer Motor (FMA-M)	End of treatment	14	35.1	21.4	0.52
	1-year follow-up	14	35.9	20.3	
Fugl–Meyer Sensory (FMA-S)	End of treatment	11	8.5	4.1	0.56
	1-year follow-up	11	8.2	3.8	
Fugl–Meyer Range of Motion (FMA-ROM)	End of treatment	10	19.8	3.5	0.60
	1-year follow-up	10	20.1	3.0	
Fugl–Meyer Pain (FMA-Pain)	End of treatment	10	20.8	3.8	0.50
	1-year follow-up	10	21.3	3.8	
Shoulder Flexion (°)	End of treatment	10	86.6	78.5	0.11
	1-year follow-up	10	97.1	73.4	
Shoulder Abduction (°)	End of treatment	10	82.0	78.1	0.63
	1-year follow-up	10	87.0	71.2	
Shoulder Rotation (°)	End of treatment	10	42.9	38.7	0.16
	1-year follow-up	10	58.7	51.3	
Grip Strength (Jamar, kg)	End of treatment	9	5.8	9.0	0.86
	1-year follow-up	9	6.0	7.9	
Grip Strength (Pablo, kg)	End of treatment	9	5.1	6.8	0.88
	1-year follow-up	9	4.9	5.8	
Key Pinch Grip (kg)	End of treatment	9	2.0	3.0	0.68
	1-year follow-up	9	2.2	2.8	
Pain (VAS, 0–10)	End of treatment	13	1.4	3.2	0.37
	1-year follow-up	13	2.0	3.7	

**Table 6:** Within-phase changes in upper-extremity ROM, grip strength, and key pinch grip.

Outcome Measure	Phase	N	Before M (SD)	After M (SD)	*p*
**Shoulder Flexion °**	Baseline 1	11	69.0 (76.0)	78.4 (78.1)	0.14
	Baseline 2	15	88.4 (68.2)	88.7 (72.8)	0.95
	rTMS	14	71.2 (63.8)	78.5 (70.0)	0.34
	Robotic Therapy	15	76.9 (67.6)	84.6 (63.4)	0.23
	Intensive Training	15	84.0 (72.3)	87.1 (70.1)	0.63
**Shoulder Abduction °**	Baseline 1	11	63.4 (74.2)	57.2 (63.8)	0.54
	Baseline 2	15	84.1 (62.3)	81.7 (70.7)	0.73
	rTMS	14	52.0 (54.7)	60.7 (60.0)	**0.03**
	Robotic Therapy	15	58.8 (55.0)	69.8 (52.4)	**0.01**
	Intensive Training	15	60.3 (58.9)	74.9 (65.7)	**0.05**
**Shoulder Rotation °**	Baseline 1	10	31.8 (35.0)	32.2 (35.4)	0.87
	Baseline 2	15	36.0 (33.6)	40.5 (33.3)	0.19
	rTMS	14	32.0 (27.8)	33.3 (30.3)	0.72
	Robotic Therapy	15	34.6 (30.3)	36.2 (27.8)	0.50
	Intensive Training	15	39.6 (31.3)	36.3 (33.7)	0.38
**Elbow Flexion °**	Baseline 1	10	90.5 (51.2)	88.4 (48.9	0.40
	Baseline 2	14	105.8 (36.8)	106.3 (35.2)	0.88
	rTMS	13	95.5 (39.6)	94.6 (46.3)	0.89
	Robotic Therapy	15	93.8 (48.4)	100.7 (38.5)	0.36
	Intensive Training	14	98.2 (39.3)	104.5 (37.9)	0.21
**Wrist Dorsal Flexion °**	Baseline 1	11	21.4 (28.2)	22.3 (28.9)	0.34
	Baseline 2	16	31.6 (28.1)	31.9 (29.5)	0.85
	rTMS	15	23.0 (27.4)	27.0 (29.5	0.11
	Robotic Therapy	16	27.8 (27.1)	30.9 (27.8)	0.16
	Intensive Training	16	25.3 (26.3)	28.4 (29.1)	0.24
**Wrist Palmar Flexion °**	Baseline 1	11	25.9 (30.8)	25.0 (30.8)	0.55
	Baseline 2	16	40.13 (29.5)	37.9 (27.7)	**0.05**
	rTMS	15	31.7 (29.0)	33.3 (26.6)	0.59
	Robotic Therapy	16	36.6 (27.4)	38.6 (26.3)	0.33
	Intensive Training	16	34.1 (23.8)	39.7 (29.1)	0.10
**Grip Strength–Jamar**	Baseline 1	12	3.7 (6.6)	4.2 (5.8)	0.54
Kg	Baseline 2	16	6.2 (6.7)	6.5 (7.8)	0.77
	rTMS	15	5.7 (6.6)	5.0 (6.0)	0.60
	Robotic Therapy	16	4.2 (5.7)	6.3 (6.8)	**0.04**
	Intensive Training	16	4.9 (6.0)	5.3 (6.4)	0.44
**Key Pinch Grip (kg)**	Baseline 1	12	1.1 (2.0)	1.4 (1.9)	0.88
	Baseline 2	16	2.1 (2.1)	2.7 (2.7)	**0.03**
	rTMS	14	1.4 (1.9)	1.2 (1.9)	0.47
	Robotic Therapy	16	1.9 (2.3)	1.9 (2.0)	0.95
	Intensive Training	16	1.6 (2.0)	2.2 (2.3)	**0.02**
**Pain (NRS)**	Baseline 1	10	1.3 (3.1)	1.1 (1.9)	0.88
	Baseline 2	14	2.1 (3.4)	2.5 (4.0)	0.34
	rTMS	13	0.6 (1.3)	1.1 (1.7)	0.07
	Robotic Therapy	13	1.9 (3.6)	1.4 (3.2)	0.30
	Intensive Training	14	1.6 (2.8)	1.60 (3.3)	0.83

## Data Availability

The datasets generated and/or analyzed during the current study are not publicly available due to the inclusion of identifiable clinical data from hospital patients and applicable data protection regulations. De-identified data may be made available by the corresponding author upon reasonable request and subject to approval by the relevant institutional and ethical authorities. Correspondence concerning data access should be addressed to hannuheikkila100@gmail.com.
